# Anaphylaxis

**DOI:** 10.1186/s13223-024-00926-3

**Published:** 2024-12-09

**Authors:** Elissa M. Abrams, Waleed Alqurashi, David A. Fischer, Timothy K. Vander Leek, Anne K. Ellis

**Affiliations:** 1 Division of Allergy, Department of Pediatrics, University of British Columbia, BC Children’s Hospital, Vancouver, BC Canada; 2 Department of Pediatrics, Section of Allergy and Clinical Immunology, University of Manitoba, Winnipeg, MB Canada; 3 Department of Pediatrics and Emergency Medicine, University of Ottawa, Ottawa, ON Canada; 4 Division of Allergy, Department of Internal Medicine, Western University, London, ON Canada; 5 Department of Pediatrics, Faculty of Medicine & Dentistry, University of Alberta, Edmonton, AB Canada; 6 Division of Allergy & Immunology, Department of Medicine, Queen’s University, Kingston, ON Canada

**Keywords:** Anaphylaxis, Diagnosis, Acute management, Epinephrine, Long-term management, Anaphylaxis emergency plan

## Abstract

Anaphylaxis is an acute, potentially fatal systemic hypersensitivity reaction with varied mechanisms and clinical presentations. Although prompt recognition and treatment of anaphylaxis are imperative, both patients and healthcare professionals often fail to recognize and diagnose its early signs. Clinical manifestations vary widely, however, the most common signs are cutaneous symptoms, including urticaria and angioedema. Immediate intramuscular administration of epinephrine into the anterolateral thigh is first-line therapy, and is always safe even if the diagnosis is uncertain. The mainstays of long-term management include specialist assessment, allergen avoidance measures, and the provision of an epinephrine auto-injector with an individualized anaphylaxis emergency plan. This article provides an overview of the causes, clinical features, diagnosis, and acute as well as long-term management of anaphylaxis.

## Key take-home messages

• Anaphylaxis is the most severe form of a hypersensitivity reaction that is rapid in onset and potentially fatal.

• Prompt recognition and treatment are critical in anaphylaxis.

• The diagnosis is based primarily on clinical signs and symptoms.

• The most common clinical manifestations are cutaneous symptoms, including urticaria, facial/lip swelling, erythema, and pruritus.

• Referral to an allergist or immunologist should be considered for all persons who have experienced a previous anaphylactic episode.

• Epinephrine is the drug of choice for anaphylaxis and should be given immediately, even if the diagnosis is uncertain; intramuscular administration into the anterolateral thigh is recommended.

• There are no absolute contraindications to the use of epinephrine.

• Up to 15% of anaphylaxis cases will have a biphasic response, with a second wave of symptomatology.

• The mainstays of long-term treatment include: specialist assessment, avoidance measures, the provision of an epinephrine auto-injector and an individualized anaphylaxis emergency plan.

## Introduction

Anaphylaxis is a serious systemic hypersensitivity reaction that is rapid in onset and potentially life-threatening [1]. While the prevalence of anaphylaxis is estimated to be as high as 2% and appears to be increasing, the fatality rate is extremely low (i.e., < 0.0001% prevalence in the general population, or < 0.5% case fatality rate in those hospitalized or presenting to the emergency department [ED] with anaphylaxis) and appears to be decreasing [2–6].

Globally, ED visits for anaphylaxis are increasing [6]. According to data from the Canadian Institute for Health Information (CIHI), the rate of children visiting Ontario and Alberta EDs for anaphylaxis more than doubled between 2007 and 2014 [7]. Among those aged 13–17 years, ED visits increased significantly, from 23 per 100,000 in 2007 to 59 per 100,000 in 2014. The highest annual rate of ED visits was among children aged 4 years and younger. The Cross-Canada Anaphylaxis Registry (C-CARE) also reported a steady increase in paediatric ED visits, from 1.8 per 1000 in 2011 to 4.5 per 1000 in 2015 [8].

Prompt recognition and treatment with intramuscular epinephrine is the most effective means at reducing fatalities from anaphylaxis. However, anaphylaxis is often under-recognized and treated inadequately. The rapid onset and resolution of mild anaphylaxis contribute to under-diagnosis and inappropriate treatment. Furthermore, there is no single test to diagnose anaphylaxis in routine clinical practice. This article will provide an overview of the causes, modulating factors and clinical features of anaphylaxis as well as strategies for the accurate diagnosis and management of the condition.

## Causes & modulating factors

Most episodes of anaphylaxis are triggered through an immunologic mechanism involving immunoglobulin E (IgE), which leads to mast cell and basophil activation/degranulation and the subsequent release of inflammatory mediators such as histamine, platelet-activating factor, leukotrienes, tryptase and prostaglandins. The most common causes of IgE-mediated anaphylaxis are foods, medications, and stinging insects (see Table 1) [9]. In children, anaphylaxis is most often caused by foods, while venom- and drug-induced anaphylaxis is more common in adults [10–13].

**Table 1:** Causes and modulating factors of anaphylaxis

**Causes of anaphylaxis**
**Common**: • Foods: most commonly peanuts, tree nuts, egg, fish, shellfish, cow’s milk, and wheat • Medications: most commonly antibiotics • Insect stings (bees and wasps) • Unidentified (no cause found; idiopathic anaphylaxis) **Less common**: • Natural rubber latex • Semen • Allergen immunotherapy: SCIT and OIT • Transfusions • Topical medications (e.g., chlorhexidine, Polysporin)
**Modulating factors of anaphylaxis**
**Intrinsic**: • Genetic predisposition (e.g., HLA-DR) • Comorbidities (e.g., poorly-controlled asthma, clonal MC disease, hereditary alpha-tryptasemia) • Biologic factors (e.g., hormonal fluctuations, age, PAF level and function) **Extrinsic**: • Physical activity • Alcohol • Infections • Drugs (e.g., beta-blocker, ACE inhibitors, NSAIDs) • Emotional stress

ACE: angiotensin-converting enzyme; HLA-DR: human leukocyte antigen DR genotype; MC: mast cell; NSAIDs: non-steroidal anti-inflammatory drugs; OIT: oral immunotherapy; PAF: platelet-activating factor; SCIT: subcutaneous immunotherapy

The severity of anaphylaxis can be further modulated by certain intrinsic (e.g., genetic predisposition, co-morbidities, hormonal fluctuations) and extrinsic factors, which include co-factors such as exercise, alcohol, acute infections and emotional stress (see Table 1) [14–16]. Co-factors can influence reactions in two ways: (1) by increasing severity such that individuals have only mild symptoms in the absence of the cofactor, but a more severe reaction when the cofactor is present, or (2) by reducing the reaction threshold (i.e., the dose needed to trigger any symptoms) so that patients have no symptoms in the absence of the cofactor and only react with the cofactor present [15]. For example, in food-dependent exercise-induced anaphylaxis, food and exercise are each tolerated separately, but onset of anaphylaxis occurs during or soon after physical exercise preceded by ingestion of a causative food (most commonly wheat) [17]. Cofactors reportedly play a role in approximately 30% of anaphylactic reactions in adults and 14–18% in children [16].

Co-morbidities and medications may also affect the severity of anaphylactic reactions and patient response to treatment. For example, patients with poorly controlled asthma and cardiovascular disease are more likely to experience a poor outcome from anaphylaxis. Concurrent administration of beta-blockers can interfere with the patient’s ability to respond to epinephrine, the first line of treatment for anaphylaxis (discussed later). Additionally, the use of angiotensin-converting enzyme (ACE) inhibitors may impact a patient’s compensatory physiologic response to anaphylaxis, leading to more severe reactions, although evidence is conflicting [9, 10]. In fact, some reports suggest that the use of any antihypertensive medication may worsen an anaphylactic reaction [18].

## Signs and symptoms

As anaphylaxis is a generalized systemic reaction, a wide variety of clinical signs and symptoms involving the skin, gastrointestinal, respiratory, cardiovascular and neurological systems can be observed (see Table 2). The most common clinical manifestations are cutaneous symptoms, including urticaria (hives), angioedema (swelling), erythema (flushing), and pruritus (itching) [19–21], followed by respiratory and then gastrointestinal symptoms. Central nervous system symptoms (e.g., excessive irritability in young children; *angor animi* [an impending sense of doom] in adults) can also occur. Death due to anaphylaxis usually occurs because of respiratory failure and/or cardiovascular collapse. It is important to note that the signs and symptoms of anaphylaxis are unpredictable and may vary from patient to patient and from one reaction to another. For example, 10–20% of anaphylaxis cases do not have skin involvement and, in infants, anaphylaxis can be subtle with primarily behavioural changes (i.e., excessive irritability or lethargy) [21]. Therefore, the absence of one or more of the common symptoms listed in Table 2 does not rule out anaphylaxis and should not delay immediate treatment as timely epinephrine is both life-saving and safe.

**Table 2:** Signs and symptoms of anaphylaxis

**Skin** • Urticaria • Facial/lip swelling • Erythema (flushing) • Pruritus	**Gastrointestinal**: • Nausea • Vomiting • Abdominal pain • Diarrhea
**Respiratory**: • *Upper airway*: — Nasal congestion/rhinorrhea — Sneezing — Hoarseness — Cough — Stridor — Laryngeal edema • *Lower airway*: — Dyspnea — Cough — Bronchospasm — Wheezing — Chest tightness — Cyanosis/hypoxia	**Neurologic**: • Light-headedness • Dizziness • Confusion • Incontinence **Oropharyngeal**: • Pruritus, tingling • Angioedema **Other**: • Sense of impending doom • Anxiety • Irritability/lethargy (infancy)
**Cardiovascular**: • Hypotension • Dizziness • Syncope • Tachycardia • Wide pulse pressure	

The signs and symptoms of anaphylaxis typically develop within minutes after exposure to the offending antigen, but may occasionally occur as late as 2 h post exposure (and up to 4 h after red meat ingestion in the case of alpha-gal syndrome) [22]. Symptoms usually follow a uniphasic course, with resolution of symptoms within hours of treatment. However, between 0.4 and 15% of reactions follow a biphasic course [23], which is characterized by an asymptomatic period of at least 1 h (up to 48 h) followed by recurrent or new symptoms not caused by antigen re-exposure [24].

## Diagnosis

The diagnosis of anaphylaxis during an acute episode is based primarily on clinical signs and symptoms. Following the acute episode, confirmation of the diagnosis requires a detailed description of the acute episode, including antecedent activities and events. The diagnostic criteria for anaphylaxis are shown in Table 3 [21]. Since confirming the diagnosis and etiology of anaphylaxis is often complex, referral to an allergist with training and expertise in the identification and management of anaphylaxis is strongly encouraged.

**Table 3:** Clinical criteria for diagnosing anaphylaxis [21]

Anaphylaxis is highly likely when *any* one of the following 2 criteria are fulfilled:	
**1**	**Acute onset of an illness** (minutes to several hours) **with simultaneous involvement of the skin**,** mucosal tissue**,** or both** (e.g., generalized hives, pruritus or flushing, swollen lips-tongue-uvula) **and at least 1 of the following**: a. **Respiratory compromise** (e.g., dyspnea, wheeze-bronchospasm, stridor, reduced PEF, hypoxemia) b. **Reduced BP** or associated symptoms of end-organ dysfunction (e.g., hypotonia [collapse], syncope, incontinence) c. **Severe GI symptoms** (e.g., severe crampy abdominal pain, repetitive vomiting), especially after exposure to non-food allergens
**2**	**Acute onset of hypotension**^a^** or bronchospasm**^b^** or laryngeal involvement**^**c**^** after exposure to a known or highly probable allergen**^**d**^** for that patient (minutes to several hours)**,** even in the absence of typical skin involvement**

PEF = Peak expiratory flow; BP: blood pressure; GI: gastrointestinal

^a^ Hypotension defined as a decrease in systolic BP greater than 30% from that person’s baseline, OR (i) Infants and children under 10 years: systolic BP less than (70 mmHg + [2 x age in years]) (ii) Adults and children over 10 years: systolic BP less than < 90 mmHg

^b^ Excluding lower respiratory symptoms triggered by common inhalant allergens or food allergens perceived to cause “inhalational” reactions in the absence of ingestion

^c^ Laryngeal symptoms include: stridor, vocal changes, odynophagia

^d^ An allergen is a substance (usually a protein) capable of triggering an immune response that can result in an allergic reaction. Most allergens act through an IgE-mediated pathway, but some non-allergen triggers can act independent of IgE (for example, via direct activation of mast cells)

### History

The clinical history is the most important tool to establish the cause of anaphylaxis and must take precedence over diagnostic tests. It should elicit information about clinical manifestations, potential triggers encountered immediately prior to the onset of the reaction (e.g., foods, medications or insect bites/stings), as well as the patient’s activities preceding the event (e.g., exercise, alcohol use) and other potential co-factors (see earlier discussion on co-factors).

### Diagnostic tests

The diagnosis of a specific cause of anaphylaxis may be supported by the results of skin tests and/or in vitro IgE tests [10]. These tests can determine the presence of specific IgE antibodies to foods, medications (e.g., penicillin), and stinging insects. However, for the majority of medications, standardized skin tests and/or in vitro tests are not available.

The documentation of elevated concentrations of mast cell and basophil mediators such as plasma histamine or serum or plasma total tryptase can sometimes support the clinical diagnosis of anaphylaxis. It is critical to obtain blood samples for these measurements as soon as possible after the onset of symptoms since elevations are transient. Anaphylaxis phenotypes in which a baseline tryptase might be most helpful include idiopathic anaphylactic shock and idiopathic, drug-induced, and insect-sting anaphylaxis. However, anaphylaxis can occur even with a normal tryptase level.

### Differential diagnosis

Other diagnoses that might present with signs and/or symptoms characteristic of anaphylaxis should be excluded [21]. Table 4 summarizes some of the most common conditions that mimic anaphylaxis and that need to be considered in the differential diagnosis. Recurrent episodes of anaphylaxis may suggest underlying systemic mastocytosis or mast cell activation syndrome.

**Table 4:** Differential diagnosis of anaphylaxis

• Foreign body aspiration • Vasovagal reactions (characterized by hypotension, pallor, bradycardia, weakness, nausea and vomiting) • Vocal cord dysfunction, (i.e., paradoxical vocal fold motion) • Severe acute asthma • Pulmonary embolism • Acute anxiety (e.g., panic attack or hyperventilation syndrome) • Myocardial dysfunction • Acute poisoning • Hypoglycemia • Seizures	

## Treatment

### Acute management

Epinephrine is the drug of choice for anaphylaxis and should be given immediately to any patient with suspected anaphylaxis, together with a rapid assessment of circulation and breathing [21, 25]. Treatment should be provided even if the diagnosis is uncertain since there is no contraindication to the use of epinephrine.

The recommended dose of epinephrine for anaphylaxis is 0.01 mg/kg (maximum 0.5 mg) administered intramuscularly every 5–15 min as necessary [21, 25]. Intramuscular administration into the anterolateral thigh is recommended as it allows for more rapid absorption and higher plasma epinephrine levels compared to subcutaneous or intramuscular administration in the upper arm [26, 27]. Glucagon could also be considered in patients using beta-blockers or in those who are pregnant.

Given that early administration of epinephrine is the only life-saving intervention available for anaphylaxis and is effective if administered promptly and correctly, the Canadian Society of Allergy and Clinical Immunology (CSACI) recently released a statement on considerations for at-home management of anaphylaxis with epinephrine use [28]. According to the CSACI, immediate at-home management with epinephrine administration, without emergency medical service (EMS) activation, is appropriate in the following stringent set of circumstances:

1. Patient/caregiver comfort level with the recognition and management of anaphylaxis, in particular the prompt and correct use of epinephrine auto-injectors.
2. Immediate access to at least two, in date, weight-appropriate dose of epinephrine autoinjectors.
3. Absence of risk factors for a biphasic reaction: a prior biphasic reaction, a moderate-to-severe reaction, delayed use of epinephrine (> 60 min) or requirement of more than one dose of epinephrine.
4. Absence of risk factors for severe anaphylaxis outcomes: cardiovascular disease, asthma (especially active or poorly controlled), or mastocytosis.
5. Symptom resolution with one dose of epinephrine administration.
6. Patient/caregiver preference.

In all other circumstances, EMS should continue to be activated, and patients must be transported to a hospital for evaluation and observation. Ideally, patients should be placed in a supine position, unless vomiting or having respiratory distress, to prevent or to counteract potential circulatory collapse. Pregnant patients should be placed on their left side [10]. Once supine, patients should not be allowed to sit up until clearly fully stabilized, owing to the risk of ‘empty ventricle syndrome’, which can precipitate a profound loss of blood pressure and death [29].

As mentioned earlier, patients with poorly controlled asthma are at increased risk of a fatal reaction. In these patients, anaphylaxis may be mistaken for an asthma exacerbation and inappropriately treated solely with asthma inhalers. Therefore, if acute asthma symptoms occur in an individual with suspected anaphylaxis, epinephrine should be given along with inhaled beta_2_-agonists [20].

Beta_2_-agonists may also be considered in anaphylaxis patients with persistent signs of lower airway obstruction despite intramuscular epinephrine. Oxygen therapy should also be considered in any patient with respiratory distress or anaphylactic shock. An intravenous bolus of crystalloid or balanced fluid solutions should also be administered for patients with shock, hypotension, or persistent abdominal pain or vomiting despite 1–2 doses of epinephrine since massive fluid shifts can occur rapidly in anaphylaxis due to increased vascular permeability [30–32]. Volume replacement is particularly important for patients who have persistent hypotension despite epinephrine injections.

Other adjunctive therapies, such as antihistamines and corticosteroids, have limited benefits in anaphylaxis. Therefore, they should never replace or delay epinephrine administration. Non-sedating antihistamines could be considered in patients with persistent cutaneous symptoms despite epinephrine administration. However, sedating antihistamines (such as diphenhydramine and hydroxyzine) should be avoided due to the risk of drowsiness/somnolence, dizziness, orthostatic hypotension (that may mimic anaphylaxis) and fatal cardiac arrhythmias [33–35]. Although corticosteroids are often administered in the setting of anaphylaxis, recent evidence shows that they have no benefits in preventing severe or refractory reactions or reducing the risk of a biphasic reaction [21, 25, 36].

**Fig. 1:**
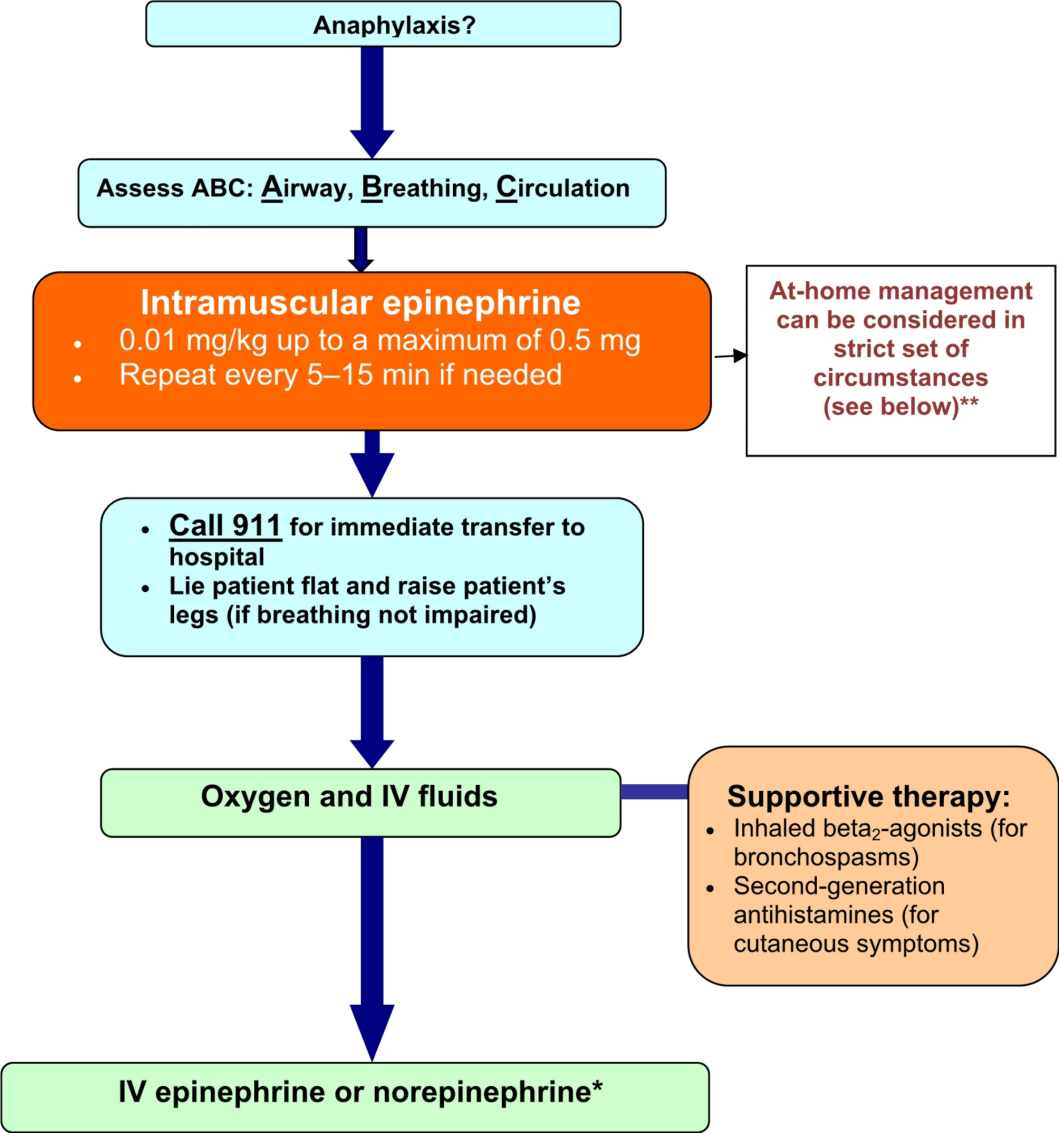
Simplified algorithm for the acute management of anaphylaxis. *Should be administered if there is no improvement after intramuscular epinephrine by a physician trained in the use of IV epinephrine with capacity for continuous blood pressure and cardiac monitoring. **At home management, without EMS activation, is appropriate in the following stringent set of circumstances: (**1**) patient/caregiver comfort level with the recognition and management of anaphylaxis, in particular the prompt and correct use of epinephrine auto-injector; (**2**) immediate access to at least two, in date, weight-appropriate dose of epinephrine autoinjectors; (**3**) absence of risk factors for a biphasic reaction: a prior biphasic reaction, a moderate-to- severe reaction, delayed use of epinephrine (> 60 min) or requirement of more than one dose of epinephrine; (**4**) absence of risk factors for severe anaphylaxis outcomes: cardiovascular disease, asthma (especially active or poorly controlled), mastocytosis; (**5**) symptom resolution with one dose of epinephrine administration; (**6**) patient/caregiver preference IV: intravenous; IM: intramuscular; EMS: emergency medical services

If anaphylaxis fails to respond to 2–3 doses of intramuscular epinephrine and intravenous fluids, an intravenous infusion of vasopressors, such as epinephrine and/or norepinephrine, may be required. However, these infusions should be given by a physician who is trained and experienced in its use and has the capacity for continuous blood pressure and cardiac monitoring. Figure 1 provides a simplified algorithm for the acute management of anaphylaxis.

Following acute treatment, patients should be observed for a period of time due to the risk of a biphasic response or possible recurrence of the reaction as epinephrine wears off. The observation period should be individualized based on the severity of the initial reaction, the presence of modulating factors, and access to epinephrine. Table 5 provides criteria that can be used as a general guide for ED patient monitoring and disposition [37].

**Table 5:** Criteria for ED patient monitoring and disposition of anaphylaxis [37]

Criteria	Disposition Plan
Mild anaphylaxis that resolved after one dose of timely epinephrine and remained asymptomatic for at least 1 h after epinephrine administration	Monitor for 2 h from the onset of the reaction
Any of the following: • Required two doses of epinephrine to treat the reaction • Presented late in evening • Lives alone or far from emergency care • Has no immediate access to epinephrine autoinjector • History of severe or currently uncontrolled asthma	Monitor for 6 h from the onset of the reaction or overnight
Any of the following: • Severe anaphylaxis (e.g., anaphylactic shock, severe respiratory distress) • Required > 2 doses of IM epinephrine • Drug-induced anaphylaxis	Admit to hospital for at least 24 h

## Long-term management

The mainstays of long-term management for patients who have experienced anaphylaxis include: specialist assessment, a prescription for an epinephrine auto-injector, patient and caregiver education on avoidance measures, and the provision of an individualized anaphylaxis action plan.

### Specialist assessment

After acute anaphylaxis, patients should be assessed for their future risk of anaphylaxis, ideally by an allergist. These specialists are experienced in identifying and confirming the cause of anaphylaxis, educating patients on appropriate avoidance strategies, drafting an anaphylaxis emergency plan, and advising whether immunotherapy is appropriate [10, 20].

### Prescription for an epinephrine auto-injector

A prescription for an epinephrine auto-injector should be provided to all patients who have experienced anaphylaxis previously, including those who have had **any** rapid-onset systemic allergic reaction (gastrointestinal, respiratory, cardiac); diffuse hives to any food or insect stings; or **any** rapid-onset (i.e., minutes to hours) reaction of any severity to the highest risk foods such as peanut, tree nuts, fish, and shellfish [20].

There are currently three epinephrine auto-injectors approved in Canada (Table 6). All three products come in two dosage forms (0.15 mg and 0.30 mg for EpiPen and Allerject, and 0.3 mg and 0.5 mg for Emerade), which are prescribed according to weight (note that at the time of publication of this manuscript, Emerade has been recalled and Allerject is difficult to access in Canada). Table 6 outlines current product monograph and CSACI weight-based dosing recommendations [38]. These devices should be stored properly (avoiding temperature extremes) and replaced before the expiration date. Note that an epinephrine nasal spray (Neffy) has been approved for the emergency treatment of anaphylaxis in the United States and may be available in Canada in the future.

**Table 6:** Available epinephrine autoinjectors in Canada with current product monograph and CSACI recommendations for dosing based on weight [38]

Brand	Available doses (mg)	Weight recommendation for dosing	
		Product monographs	CSACI
EpiPen	0.30	≥ 30 kg	≥ 25 kg
EpiPen Jr	0.15	15–30 kg	< 25 kg
Allerject*	0.30	≥ 30 kg	≥ 25 kg
Allerject*	0.15	15–30 kg	< 25 kg
Emerade*	0.5	Adult: >60 kg: 0.3 to 0.5 mg depending on clinical judgement	≥ 45 kg
Emerade*	0.3	Pediatric: >30 kg Adolescent: >30 kg (follow dosage recommendation for adult above) Adult: <60 kg	≥ 25 kg < 45 kg

*Note that at the time of publication of this manuscript, Emerade has been recalled and Allerject is difficult to access in Canada

Upon prescription of an epinephrine auto-injector, healthcare providers must instruct the patient on how and when to use the device. Instructions on proper use should be reviewed verbally and accompanied by website links and/or written material, and should be reinforced annually. For example, Food Allergy Canada recently developed a multilingual and easy-to-understand illustrated guide for patients and caregivers that can be used in office and hospital settings (see Tools and Downloads section at https://foodallergycanada.ca).

The currently available epinephrine auto-injectors have needle lengths of approximately 13 mm and 15 mm for the 0.15 mg and 0.30 mg doses, respectively. Evidence suggests that, at these needle lengths, children weighing less than 15 kg are at increased risk of injection into the bone [39] and adult females are at increased risk of subcutaneous injection [40]. Therefore, special counseling on appropriate epinephrine administration in these patients may be needed.

### Recognition of co-factors and education on avoidance measures

Patients and caregivers should be educated on certain co-factors (see earlier discussion and Table 1) that can lead to an increasingly severe anaphylactic reaction, such as exercise, alcohol use, hormonal changes (e.g., menses in women), concomitant infections and certain medications. They should also be educated about agents or exposures that may place them at risk for future reactions and should be counselled on avoidance measures that may be used to reduce the risk for such exposures. Avoidance strategies should be individualized, taking into consideration factors such as relevant triggers, age, activity, occupation, hobbies, residential conditions, and access to medical care. Individuals who have had anaphylactic reactions to foods should be instructed to read food labels carefully, watching for hidden ingredients such as “natural flavour” or “spices” that may indicate the presence of allergens (e.g., peanut, tree nuts, milk, egg, shellfish, fish, sesame, soy and wheat), as well as precautionary labelling regarding potential food allergens [10]. Guidelines and multiple randomized control trials have confirmed that oral immunotherapy (OIT) is often effective in inducing desensitization to various food allergens [41–43]. In OIT, a specialist administers a food allergen in multiple, gradually escalating doses below the patient’s reactivity threshold while supervising them for signs and symptoms of a reaction. For a more comprehensive review on OIT, please see the *Oral Immunotherapy* article in this supplement.

Patients with anaphylaxis to medications should be informed about all cross-reacting medications that should be avoided. Should there be a future essential indication for the use of the medication causing anaphylactic reactions, it may be helpful to educate patients about possible management options, such as the use of low osmolarity agents in patients with a history of reactions to radiographic contrast media or induction of drug tolerance procedures (also known as drug desensitization) [10, 25]. Induction of drug tolerance procedures temporarily modify a patient’s immunologic or non-immunologic response to a drug through the administration of incremental doses of the drug. However, drug tolerance is usually maintained only as long as the drug is administered; therefore, the procedure needs to be repeated in the future if the patient requires the drug again after finishing a prior therapeutic course. For more information on drug desensitization, please see the *Drug Allergy* article in this supplement.

Patients who have had an anaphylactic reaction to an insect sting should be advised about avoidance measures to reduce the risk of future stings. Such measures include: being alert when eating outdoors (as wasps are attracted to food), wearing shoes and long pants when in fields, and having nests or hives near the patient’s home removed [44]. More importantly, however, patients who have previously experienced venom-induced anaphylaxis are often candidates for venom immunotherapy, which is successful in preventing anaphylaxis in up to 98% of patients (see article on *Allergen Immunotherapy* in this supplement), and all should be referred for an allergy assessment.

Patients should also obtain and wear medical identification (such as a MedicAlert bracelet/necklace) that indicates that they have experienced anaphylaxis as well as the responsible agent. Patients should also be instructed to avoid drugs that might increase their susceptibility and/or complicate the management of an anaphylactic event, such as beta-blockers or ACE inhibitors [10]. However, this should be considered in a process of shared decision-making between the patient, allergist, and the prescribing physician [25].

### Anaphylaxis emergency plan

A comprehensive, individualized anaphylaxis emergency plan (also referred to as an anaphylaxis action plan) should be prepared which defines roles and responsibilities and emergency protocols. Important information that should be included in this plan is shown in Table 7 [20, 45]. Examples of such a plan, along with other relevant information and materials, are available through Food Allergy Canada [46], Translating Emergency Knowledge for Kids (TREKK) [47] and the Australasian Society of Clinical Immunology and Allergy (ASCIA) [48]. Emergency plans should be reviewed annually and updated if necessary. A copy of the plan should be made available to all relevant people, such as day-care providers, teachers, and employers. Recommendations for the management of anaphylaxis in schools and other community settings are available through Food Allergy Canada [49] or the Canadian Society for Allergy & Clinical Immunology (CSACI) [50].

**Table 7:** Components of an anaphylaxis emergency plan

**Contact details** • Names and contact details for emergencies, including family members, allergist/immunologist and primary care provider • Contact details for local emergency or ambulance services.
**Allergens/triggers** • Clear identification of allergens/triggers to be avoided: — Include generic and proprietary names of drugs and possible cross-sensitivities, if relevant
**How to recognize the signs and symptoms of anaphylaxis** • Mouth: itching, swelling of lips/tongue • Throat: itching, tightness, closure, hoarseness • Skin: itching, hives, eczema, swelling, flushing • Gut: vomiting, diarrhea, abdominal pain • Lung: shortness of breath, cough, wheeze • Heart: hypotension, dizziness, syncope, tachycardia • Neuro (or head): light-headedness • Other: feeling of impending doom, anxiety
**Medications prescribed and when they should be used** • Epinephrine auto-injectors (first line); should include detailed instructions (with photographs, if possible) on how to correctly administer the auto-injector device (for daycare, school and/or office staff) • Second-generation antihistamines (for cutaneous symptoms) • Inhaled beta_2_-agonists (for bronchospasm)
**Where medication is stored at home**,** work or school**

Adapted from: Waserman 2010 [20]; Muraro 2007 [45]

## Conclusions

Anaphylaxis is an acute, potentially fatal systemic reaction with varied mechanisms and clinical presentations. Prompt recognition and treatment of anaphylaxis are imperative; however, both patients and healthcare professionals often fail to recognize and diagnose anaphylaxis in its early stages. Diagnostic criteria which consider the variable clinical manifestations of anaphylaxis are now available and can assist healthcare providers in the early recognition of the condition. Immediate intramuscular administration of epinephrine into the anterolateral thigh is the first-line therapy for anaphylaxis, and is always safe, even if the diagnosis is uncertain. Acute management may also involve oxygen therapy, intravenous fluids, and adjunctive second-line therapies such as second-generation antihistamines or inhaled beta_2_-agonists. The mainstays of long-term management include specialist assessment, a prescription for an epinephrine auto-injector, patient and caregiver education on avoidance measures, and the provision of an individualized anaphylaxis emergency plan.

## Data Availability

Data sharing not applicable to this article as no datasets were generated or analyzed during the development of this review.

## Declarations

### About this supplement

This article has been published as part of Allergy, Asthma & Clinical Immunology, Volume 20 Supplement 03, 2024: Practical Guide for Allergy and Immunology in Canada 2024. The full contents of the supplement are available at https://aacijournal.biomedcentral.com/articles/supplements/volume-20-supplement-3.

## Abbreviations

ACE: Angiotensin-converting enzyme
ASCIA: Australasian society of clinical immunology and allergy
C-CARE: Cross-canada anaphylaxis registry
CIHI: Canadian institute for health information
CSACI: Canadian society of allergy and clinical immunology ed: emergency department
IgE: Immunoglobulin E
NSAID: Non-steroidal anti-inflammatory drugs
TREKK: Translating emergency knowledge for kids (TREKK)
